# Bibliography

**Published:** 1898-01

**Authors:** 


### Bibliography.

TIN FOIL AND ITS COMBINATIONS FOR FILLING TEETH. By Henry L. Ambler, M.S., D.D.S., M.D., Professor of Operative Dentistry and Dental Hygiene in the Dental Department of Western Reserve University, etc. Philadelphia: The S. S. White Dental Manufacturing Company; London: Claudius Ash & Sons Limited, 1897.

This is a small volume of one hundred pages on the history of the use of tin in dentistry, methods of use, and value as a fillingmaterial. It requires some courage in this day of amalgam using and experimentation for one to come forward and advocate the use of a metal that occupied a quite different relation to amalgam forty years ago than it does at the present time. In other words, amalgam then was regarded as an abomination, and tin was held next to gold in preservative value. That these conditions have been reversed does not by any means prove that the dentist of the year 1897 is correct in his judgment or practice.

The present writer can have naught but commendation for this labor of love on the part of Dr. Ambler, for the best of reasons that he has been a devoted adherent of tin as a filling-material since he began the practice of dentistry.

Dr. Ambler has been at great labor to compile the views of practitioners in the past, for the accomplishment of which, Dr. McKellops, of St. Louis, very kindly gave him the free use of his dental library, probably ranking first in this country, if not in the world. These references carry the use of tin as a filling-material back to 1783, but it was probably used anteriorly to that date.

It is satisfactory to find the author packs his tin on the cohesive principle, the only method in use by the writer for over thirty years; but if he would use a slightly heavier foil than that usually selected and make tapes without folding, it is thought better results could be attained.

Dr. Ambler insists on absolute dryness. While this is correct for all metals used in the mouth, it does not alter the fact that tin can be packed upon the cohesive principle under water. This quality, for it is a peculiarity in its working properties, becomes of great value in filling children's teeth where the rubber dam or the napkin is an impossibility. Where this is done all parts must be submerged in water.

This little book is cordially welcomed, not because it contains anything new, for it does not, but because it carries with it the hope that one of the most valuable filling-materials will not die out of practice so long as such an enthusiastic defender is left to earnestly urge its good qualities upon an unbelieving profession.

